# Does Probability Guided Hysteroscopy Reduce Costs in Women Investigated for Postmenopausal Bleeding?

**DOI:** 10.1155/2015/605312

**Published:** 2015-02-16

**Authors:** M. C. Breijer, N. van Hanegem, N. C. M. Visser, R. H. M. Verheijen, B. W. J. Mol, J. M. A. Pijnenborg, B. C. Opmeer, A. Timmermans

**Affiliations:** ^1^Department of Obstetrics and Gynecology, Academic Medical Center, University of Amsterdam, Meibergdreef 9, 1105 AZ Amsterdam, Netherlands; ^2^Department of Obstetrics and Gynecology, Erasmus Medical Center, 's Gravendijkwal 230, 3015 CE Rotterdam, Netherlands; ^3^Department of Obstetrics and Gynecology, Maastricht University Medical Center, P. Debyelaan 25, 6229 HX Maastricht, Netherlands; ^4^Department of Pathology, Radboud University Medical Center, Geert Grooteplein-Zuid 10, 6525 GA Nijmegen, Netherlands; ^5^Division of Women and Baby, Gynecological Oncology, University Medical Center, Heidelberglaan 100, 3584 CX Utrecht, Netherlands; ^6^The Robinson Institute, School of Paediatrics and Reproductive Health, The University of Adelaide, Level 3, Medical School South, Frome Road, Adelaide, SA 5005, Australia; ^7^Department of Obstetrics and Gynecology, TweeSteden Hospital, Dr. Deelenlaan 5, 5042 AD Tilburg, Netherlands; ^8^Clinical Research Unit, Academic Medical Center, University of Amsterdam, Meibergdreef 9, 1105 AZ Amsterdam, Netherlands

## Abstract

*Objective*. To evaluate whether a model to predict a failed endometrial biopsy in women with postmenopausal bleeding (PMB) and a thickened endometrium can reduce costs without compromising diagnostic accuracy. *Design, Setting, and Population*. Model based cost-minimization analysis. *Methods*. A decision analytic model was designed to compare two diagnostic strategies for women with PMB: (I) attempting office endometrial biopsy and performing outpatient hysteroscopy after failed biopsy and (II) predicted probability of a failed endometrial biopsy based on patient characteristics to guide the decision for endometrial biopsy or immediate hysteroscopy. Robustness of assumptions regarding costs was evaluated in sensitivity analyses. *Main Outcome Measures*. Costs for the different strategies. *Results*. At different cut-offs for the predicted probability of failure of an endometrial biopsy, strategy I was generally less expensive than strategy II. The costs for strategy I were always € 460; the costs for strategy II varied between € 457 and € 475. At a 65% cut-off, a possible saving of € 3 per woman could be achieved. *Conclusions*. Individualizing the decision to perform an endometrial biopsy or immediate hysteroscopy in women presenting with postmenopausal bleeding based on patient characteristics does not increase the efficiency of the diagnostic work-up.

## 1. Introduction

Postmenopausal bleeding (PMB) is the most common presenting symptom of endometrial cancer and warrants further investigation [1]. Since the 1990s, endometrial thickness measured by transvaginal ultrasound was introduced to select women for further invasive diagnostic testing to detect or rule out endometrial cancer [2–4]. Although the optimal endometrial thickness cut-off for women with PMB still remains questionable, at present most guidelines advise an endometrial thickness cut-off of 4 or 5 mm to select patients for further histological verification [1, 5–10]. Outpatient endometrial biopsy is the least invasive technique to obtain material for histological assessment. Pipelle endometrial biopsy (Pipelle de Cornier, Paris, France) is the most accurate endometrial sampling device to detect endometrial carcinoma and endometrial hyperplasia in patients with PMB [11]. Furthermore, a strategy with endometrial biopsy after endometrial thickness measurement is the most cost-effective diagnostic strategy for patients with PMB [12].

Although endometrial biopsy is the most accurate and frequently used diagnostic procedure, it has some major drawbacks in clinical practice. In 12–21% of cases, endometrial sampling fails due to technical reasons [13–15] and in 7–68% of cases the number of tissue obtained is insufficient for a reliable histological diagnosis [13–17]. Because an endometrial (pre)malignancy is present in 6–23% of the women with a failed endometrial biopsy, these patients cannot be reassured without further more invasive investigations [14, 15].

In a previous publication, we described a multivariable prediction model to predict the probability of a failed endometrial biopsy in women with PMB [15]. The purpose of the current study was to evaluate whether this model has the potential to reduce costs for the same accuracy as the regular diagnostic testing in women with PMB through a cost-minimization analysis.

## 2. Methods

We performed a cost-minimization analysis using a model based decision analytic approach. The aim was to evaluate whether in women with a first episode of PMB individualizing the decision to perform immediate diagnostic hysteroscopy rather than perform an endometrial biopsy in all women can decrease the costs of the diagnostic work-up. This decision was based on the probability of a failed endometrial biopsy, estimated with a clinical prediction model based on patient characteristics. Recently, we developed such a model in women presenting with postmenopausal bleeding and an endometrial thickness of more than 4.0 mm. Details on model development are presented in the original paper [15].

In short, data of 356 women with PMB were included in a multivariable regression analysis. Characteristics satisfying the criteria for inclusion in the model were time since menopause, hypertension, endometrial thickness (categorized), and nulliparity. A failed endometrial biopsy was defined as a technical failure or insufficient number of tissue for a reliable diagnosis. Endometrial biopsy failed in 44.4% (95% CI 39.3 to 49.6%) of the women (158/356). The discriminative capacity of the model was assessed with the area under the receiver operator characteristic (ROC) curve and was 0.64 (95% CI 0.58 to 0.70). The calibration of the model was good indicating that there was high agreement between the predicted probabilities and the observed proportion of failed endometrial biopsies.

### 2.1. Cost Minimization Analysis

**Figure 1:**
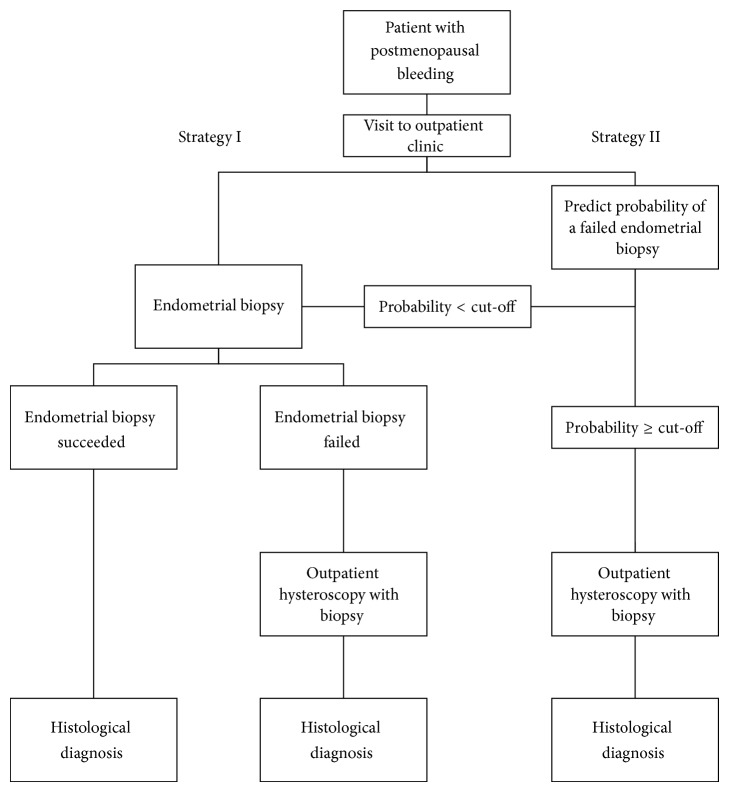
Flowchart representing the two diagnostic strategies.

The cost minimization analysis compared two diagnostic strategies: (I) attempting office endometrial biopsy in all women and performing outpatient hysteroscopy after failed biopsy and (II) decision for endometrial biopsy or direct referral to outpatient hysteroscopy based on model based probability of a failed biopsy. The diagnostic strategies are represented in Figure 1. The sensitivity and specificity associated with different failure rate cut-offs were calculated in the prospective cohort. As the individualized strategy may become cost saving at a certain cut-off, a threshold analysis was performed to identify this cut-off.

#### 2.1.1. Assumptions

Outpatient hysteroscopy with biopsy was assumed to be the gold standard with a 100% correct diagnosis. Furthermore, it was assumed that in all women diagnostic investigations would be continued until adequate material for a histological diagnosis is obtained. Hence, all women in whom endometrial biopsy failed are subsequently referred to outpatient hysteroscopy with biopsy. With both strategies, material for diagnosis will be obtained in all patients.

#### 2.1.2. Costs

The analysis was conducted from a health care provider's perspective and included direct medical costs in euros (Table 1). Unit costs were based on Dutch reference prices [18] and local cost calculations (Academic Medical Center, Amsterdam). Costs for outpatient hysteroscopy were estimated per procedure and included costs for maintenance, disinfection, and sterilization. Costs for an outpatient clinic visit included the specialist fee, costs for the assisting personnel, and overhead costs.

### 2.2. Statistical Analysis

Analyses were performed using TreeAgePro 2008 (TreeAge Software Inc., Williamstown, MA). In addition to the base-case analysis, evaluating the results for the most likely estimates of model parameters, one-way sensitivity analyses were carried out to explore the robustness of the results for uncertainty in these parameters, including model accuracy, incidence of failure, and unit cost estimates.

## 3. Results

### 3.1. Base Case Analysis

In this economic analysis, expected costs for the two diagnostic strategies were compared. As endometrial biopsy failed in 44% of the patients in the cohort study, this percentage was assumed in the assessment of strategy I. In the prospective cohort, the predicted probability of failure varied between 22 and 73%. The threshold analysis to identify a failure cut-off percentage was performed. Because in the prospective cohort the predicted probability of failure varied between 22 and 73%, we analyzed 50, 55, 60, 65, and 70% cut-offs. For these different failure probability cut-offs, sensitivity and specificity were calculated. The associated true positive rate and false negative rate for each cut-off were inserted in the cost-effectiveness model to calculate the expected costs for the two strategies and the cost differences between strategies. The results of these analyses are presented in Table 2.

In the base-case analysis, expected costs for strategy I were € 460 per patient; the costs for strategy II ranged from € 457 for a 65% cut-off to € 475 for a 55% cut-off, implicating that, only at a 65% predicted failure cut-off, strategy II appears to be less costly than strategy I. At this threshold, strategy II would result in a saving of € 3 per patient presenting with PMB (Table 2).

### 3.2. Sensitivity Analyses

All sensitivity analyses performed for cost variables to explore robustness of the results for uncertainties in cost-estimates showed results consistent with the base case analysis. For a cut-off value of 65%, the minimal expected cost-difference between strategy I and strategy II was € 1 and the maximum expected cost-difference was € 5 (Table 3). A sensitivity analysis varying the percentage of failure showed that strategy I is always cost-effective if the failure rate of endometrial biopsies is 25% or less.

## 4. Discussion

In this study a cost-minimization analysis was performed to investigate the potential for cost savings of selecting women with a first episode of PMB for endometrial biopsy or hysteroscopy based on the predicted probability of a failed biopsy. At a cut-off of 65% predicted failure rate, this possibly results in a very small saving per patient presenting with PMB.

The strength of this analysis is the new approach to the problem of a failed endometrial biopsy in women with PMB. Increasingly, prediction models are developed for clinical practice. We performed an analysis to evaluate the cost-effectiveness of the model: this is an essential step before a model can be implemented in clinical practice.

A limitation of our study is the model based approach. As for all model based studies, the validity of the model depends on the model input parameters and assumptions. We assumed that a hysteroscopy, as golden standard diagnostic procedure, would always succeed and lead to a diagnosis; in reality this is not always the case. We also assumed that an outpatient endometrial biopsy, if successful, would lead to an accurate diagnosis. In reality, the detection rate of endometrial cancer in women with postmenopausal bleeding is 99.6% for the Pipelle device (Pipelle de Cornier, Paris, France) and 97.1% for the Vabra device (Berkeley Medevices, Inc., Richmond, CA, USA) [11]. Another possible limitation is the costs used in our analyses. Because precise economic data are not available, we used the best available data that could be acquired from national and local sources [18]. To evaluate the impact of our cost estimates and ranges, we performed sensitivity analyses. The results were consistent across various analyses, implying that the conclusions are generalizable to other clinics where costs may be somewhat different. In the Netherlands, the transvaginal ultrasound is performed by the consulting gynecologist. In some other countries, a transvaginal ultrasound is a diagnostic procedure that has to be ordered separately and incurs extra costs. These differences should be taken into account when applying the results of our study to countries with a different health care system. The work-up in our model conformed to recommendations in the Dutch national guideline on the work-up for women with PMB [7].

Clark and colleagues investigated the cost-effectiveness of initial diagnostic strategies for women with PMB. Their conclusion was that, depending on the prevalence of endometrial malignancy, an endometrial biopsy after endometrial thickness measurement with a cut-off of 4 or 5 mm was the most cost-effective strategy [12]. In this analysis, however, the probability of a failed endometrial biopsy was only 12% and, in contrast to our current study, this probability was independent of patient characteristics.

Because the implementation of a prediction model in clinical practice is time consuming, the model should be easy applicable and the benefits in terms of cost-effectiveness should be substantial. In view of the results of our study, in our health care system, the potential value of using the prediction model for a failed endometrial biopsy in women presenting with PMB appears to be limited and not contributing to a more efficient use of health care resources. Based on the current study, we recommend outpatient endometrial biopsy for all women presenting with a first episode of PMB and an endometrial thickness of more than 4.0 mm. Patients in whom endometrial biopsy fails have 6–23% risk of an endometrial (pre)malignancy and cannot be reassured without further testing [14, 15]. In case of a failed endometrial biopsy, a hysteroscopy with guided biopsy should be performed. Future research should, to increase the efficiency of the diagnostic work-up for women with PMB, focus on decreasing the number of failed endometrial biopsies.

## Figures and Tables

**Table 1:** Model input: direct medical costs.

Variable	Cost (€)	Range (€)
Outpatient appointment (first visit)	129	60–250
Outpatient appointment (follow-up visit)	65	30–135
Outpatient appointment (hysteroscopy clinic visit)	194	100–400
Hysteroscopy including endometrial biopsy and pathologists fee, maintenance, and sterilization	144	70–250
Endometrial biopsy during outpatient visit including pathologists fee	89	40–150

**Table 2:** Accuracy, uncertainty, and impact of cut-off values for predicted probability of a failed endometrial biopsy in women with postmenopausal bleeding and endometrial thickness of >4.0 mm.

Cut-off failure probability (%)	Sensitivity	Specificity	Costs of strategy I (€)	Costs of strategy II (€)	Expected difference (strategy II − strategy I) (€)
50%	0.30 (0.25–0.35)	0.86 (0.81–0.91)	460	472	12
55%	0.21 (0.16–0.26)	0.92 (0.87–0.97)	460	475	15
60%	0.14 (0.09–0.19)	0.96 (0.91–1.0)	460	460	0
65%	0.075 (0.025–0.125)	0.99 (0.94–1.0)	460	457	−3
70%	0.008 (0.0–0.06)	0.99 (0.94–1.0)	460	462	2

**Table 3:** Sensitivity analysis: minimum and maximum expected costs and cost-difference for 65% cut-off value.

Variable	Strategy I		Strategy II		Difference	
	Minimum (€)	Maximum (€)	Minimum (€)	Maximum (€)	Minimum (€)	Maximum (€)
Outpatient appointment (first visit)	391	581	388	578	3	3
Outpatient appointment (follow-up visit)	409	561	408	556	1	5
Outpatient appointment (hysteroscopy clinic visit)	418	550	415	549	3	1
Hysteroscopy	427	506	424	504	3	2
Endometrial biopsy	411	521	410	516	1	5
